# A Qualitative Study of Secondary School Teachers’ Perception of Social Network Analysis Metrics in the Context of Alcohol Consumption among Adolescents

**DOI:** 10.3390/ijerph14121531

**Published:** 2017-12-08

**Authors:** Enedina Quiroga, Isaías García, José Alberto Benítez-Andrades, Carmen Benavides, Vicente Martín, Pilar Marqués-Sánchez

**Affiliations:** 1 SALBIS Research Group, Facultad de Ciencias de la Salud, Universidad de León, Campus de Ponferrada Avda/Astorga s/n, C.P. 24402 Ponferrada (León), Spain; equis@unileon.es (E.Q.); jbena@unileon.es (J.A.B.-A.); mcbenc@unileon.es (C.B.); pilar.marques@unileon.es (P.M.-S.); 2 SALBIS and SECOMUCI Research Groups, Escuela de Ingenierías Industrial e Informática, Universidad de León, Campus de Vegazana s/n, C.P. 24071 León, Spain; 3 GIGAS Research Group, Facultad de Ciencias de la Salud, Universidad de León, Campus de Vegazana s/n, C.P. 24071 León, Spain; vmars@unileon.es

**Keywords:** social network analysis, adolescence, alcohol consumption, teachers

## Abstract

Adolescence is a transitional period during which a number of changes occur. Social relationships established during this period influence adolescent behaviour and affect academic performance or alcohol consumption habits, among other issues. Teachers are very important actors in observing and guiding the evolution of their students, and should therefore have the appropriate knowledge and tools to gain insight into the complex social relationships that exist in their classes. The use of social network analysis (SNA) techniques may be helpful in order to study and monitor the evolution of these social networks. This study tries to understand how teachers perceive SNA metrics from an intuitive point of view. Using this information, useful tools could be created that allow teachers to use SNA techniques to improve their understanding of student relationships. A number of interviews with different teachers were held in secondary schools in Spain, allowing SNA concepts to be related to the everyday terms used by the teachers to characterize their students. Results from the study have an impact on questionnaire design for gathering data from students in order to perform an SNA analysis and on the design of software applications that can help teachers to understand the results of this analysis.

## 1. Introduction

Adolescence is a transitional stage in the journey toward adulthood, during which a number of conflicts and physical and mental changes arise. During this period of life, the adolescent is usually involved in a number of educational environments that play an important role in relation to the acquisition of values and attitudes that will form their identity [1]. It is also usual for students to have their first contact with drugs such as alcohol, tobacco, or cannabis during this stage [2].

Regarding the educational environment, the school and the classroom have been recognized as major variables to be taken into account when studying, preventing, and educating about substance abuse and also when planning and executing interventions [3]. School policies, classroom climate, or peer relationships play an important role in encouraging or discouraging certain behaviours.

The school and the classroom are where the adolescent establishes relationships with other partners, building a network of relationships that will also influence their personal development. Understanding how these relationships are established and how they evolve may be of great help in understanding a number of facts regarding student performance, from academic achievements to health-related behaviours such as substance consumption patterns [4,5].

Teaching staff are critical actors, especially during secondary school. Teachers are the people who have the best knowledge of how students socialize in the classroom; they know about the evolution of each of their pupils and can detect problems when they arise [6]. They also provide leadership for their students, and the behaviours that they exhibit as leaders have an impact on a variety of student outcomes [7].

Many studies stress this role of teachers and the need to train them in substance-abuse-prevention education and intervention because evidence has been found demonstrating that these programs achieve better results (in terms of both impact and sustainability over time) when teachers play a central role in their development [8]. Training of teachers regarding these issues should include providing them with techniques and tools for planning, evaluating, and monitoring the corresponding programs.

Previous experiences also suggest that programs that are student-oriented rather than substance-oriented offer superior outcomes. The use of interactive methods and small working groups provides opportunities for a better interchange of ideas and opinions, and it encourages and promotes participation while reducing egocentrism and defensive attitudes in the student [9].

Once a given intervention program is in process, it is very useful to know the situation of the group at any time, and how the different actors play different roles and establish links with others. Techniques from social network analysis [10] can be applied in order to study how students relate to their peers in the classroom and how these relationships evolve over time [11]. In fact, social network analysis has been used for a number of years as a research methodology for practical studies in community settings. The foci of these studies include program evaluation, participatory governance, health agent selection, sociogram generation and analysis, finding relevant actors in an intervention, empowerment strategies, mediation, community coalitions, and preventive message dissemination, among others [12]. In the context of alcohol consumption in adolescents, a number of studies have been carried out using social network analysis (SNA) techniques and tools for different purposes [13,14]. According to Lozares [15], social network theory analyses behaviours by studying how the actors are connected to each other in the various situations in which they are observed. In this case, the unit of analysis is the individual and the set of relationships. This theory assumes that a cause, consequence, or association between two aspects can be conceptualized as a network. This perspective fits the present research, as it allows the classroom to be conceptualized in terms of networks where students are interconnected, generating a “system” [16].

The premise behind this paper is that applying social network analysis to assist in study and intervention programs on adolescent substance consumption habits may be of great interest [17], but this process must also involve teaching staff. To this end, teachers should be provided with tools that can introduce them to SNA metrics (measures quantifying the number of connections of an actor or their role or position in the network, for example) and results without them needing to be experts in SNA. On the other hand, SNA techniques and methods must be designed and adapted to the context in which they are going to be applied, from the gathering of data to the interpretation of the results.

The study presented in this paper tries to understand how teachers perceive social network analysis concepts and metrics, and it also seeks to obtain information about how they identify and describe the relationships and the different roles that can be played by students in their classrooms. 

The results from the study will be of great help in designing questionnaires in order to obtain information that will be later processed with SNA techniques, and also in presenting the results of the SNA analysis to teachers in a way that they can easily understand.

## 2. Materials and Methods

Secondary school in Spain comprises two grades that correspond to 16-year-old and 17-year-old students. A number of interviews with different teachers were held in two secondary schools chosen by convenience sampling, involving a total of ten teachers (obtained by snowballing), four of them from the first grade and six from the second grade.

Teachers were interviewed using a discussion group technique. Two discussion groups were created in accordance with the two grades. The idea was to study and find any difference that could exist in the teachers’ opinion as a result of the grade at which they taught. Discussion Group 1 was composed of teachers involved in the first grade, while Discussion Group 2 was composed of teachers from the second grade. Each discussion group was moderated by the researcher.

Teachers signed an informed consent that stated the objective of the study, the structure and nature of the interviewing sessions (duration, recording, confidentiality and anonymity warranties, etc.) as well as the right to not answer a given question or even cease their participation at any time. Interviews with teachers were recorded using analogue tapes that were later disposed of. Field notes were taken using code names for individuals observed. Though no information obtained and presented in the paper is of a sensitive nature, care has been taken to ensure that the identity of participants cannot be guessed from this data.

An initial script was designed with a number of questions and subjects to be discussed with the teachers. Interviews were recorded and later processed; anonymization of the identities was carried out before the data was analysed.

The study was conducted by classifying the items to be considered under six categories: outdegree, indegree, incloseness, betweenness, eigenvector, friendship, and addictions. Five of these categories (outdegree, indegree, incloseness, betweenness, and eigenvector) correspond to different centrality measures commonly used in social network analysis [18]. The last two categories—friendship and addictions—were added in order to informally study how teachers perceived these concepts.

Categories from social network analysis measures were translated into their most common everyday interpretations in order to be easily understood by the teachers. Based on these interpretations, a number of questions were formulated in order to be presented during the interviews (see Table 1).

Each session lasted about forty minutes. It started with a brief introduction (given by the researcher) to SNA techniques and metrics and to their most common everyday interpretations, as shown in Table 1. No further information was given, as the objective was to capture the teachers’ intuitive interpretation of and opinion on these concepts. After this introduction, a discussion period started, in which the teachers offered their opinion on and interpretation of the concepts on a one-by-one basis, but with a great degree of flexibility. The final minutes were devoted to obtaining a general opinion from the teachers regarding the session and the items discussed.

## 3. Results

We will now present the results from the discussion group sessions, grouping the outcomes by category and following the structure in Table 1. A brief introduction to the given social network analysis (SNA) technique is also provided. Opinions obtained from all the groups are offered jointly for the sake of clarity.

### 3.1. Results Regarding Outdegree and Indegree

**Figure 1:**
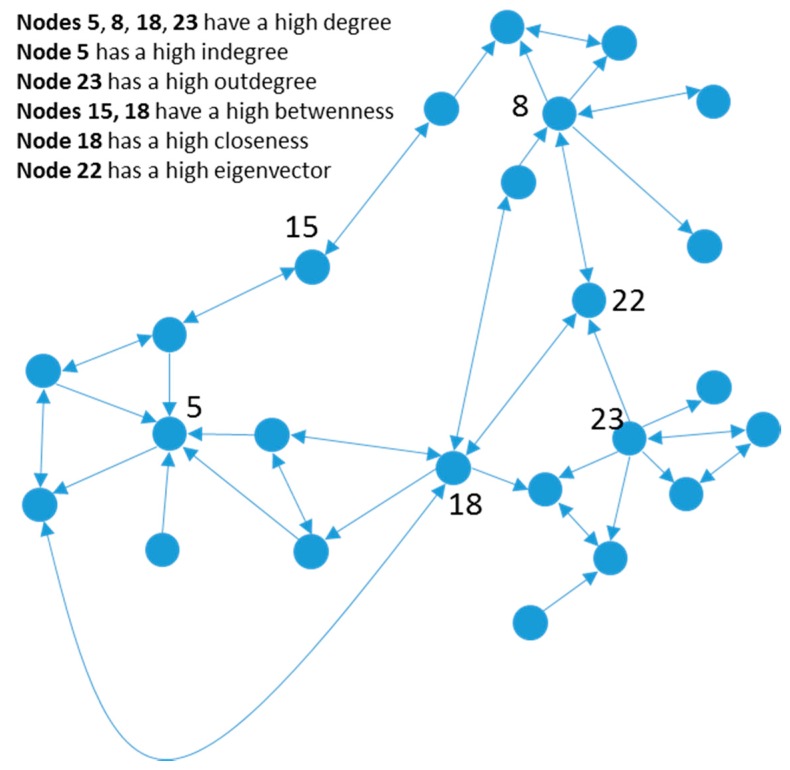
An example social network.

Degree is a social network analysis measure defined as the name of links (relationships) attached to a given node (actor) in a network [19]. This concept may be studied without taking into account the direction of the link or considering the direction of the relationship; in this latter case, the number of links entering the node is called the *indegree* of the node and the number of links leaving the node is called the *outdegree* of the node (see Figure 1). Usually, outdegree is related to sociability and approachability, while indegree is related to popularity.

Teachers agreed that there was a distinction in relation to the nature of sociability and approachability in accordance with the grade in which the students were enrolled. They indicated the importance of physical appearance in social relations when students were in the first grade of secondary school. By contrast, when they approach the final year of secondary studies and the transition to university, students tend to broaden their social circle. The number of relationships grows, the importance of physical appearance diminishes, and other factors such as academic achievement become more prominent.

When popularity (the usual interpretation of the indegree metric) was discussed, teachers tended to associate this feature with two different and rather opposing facts. Teachers from the first grade tend to assign a greater level of popularity to good-looking, well-dressed, athletic students. On the other hand, teachers from the second grade tend to perceive the most popular students to be those who exhibit a great degree of nonconformity, or even those who could be labelled as “anti-system”.

### 3.2. Results Regarding (In)Closeness

Closeness is defined in social network analysis as “the number of steps an actor must perform in order to reach another one” [20,21]. It denotes a given actor’s closeness to or farness from the rest of the components of the network.

In this case, teachers from the second grade tend to think of the “closer to the rest” students as those whose academic performance is the best, because the rest of the students can benefit from them and thus contact them frequently. Teachers from the first grade somewhat agree with this statement and point out that these “closer” students are usually not very popular themselves and so they usually seek to establish relationships with other classmates.

### 3.3. Results Regarding Betweenness

Betweenness is the number of connections passing through an actor in the path between two other actors [20,21]. Relevant actors from the point of view of betweenness usually have pro-social behaviours and so may act as mediators, facilitating contagion processes.

The teachers found it difficult to find a clear profile for students who could be considered as “pro-social” or “intermediators”. Nevertheless, they pointed out some characteristics that could facilitate such behaviour: empathy, fellowship, or cooperative spirit were cited. According to the teachers, these values are appreciated by students and interpreted by them as good for the development and cohesion of their social network.

### 3.4. Results Regarding Eigenvector

Prestige is defined in SNA as a measure for finding the most central actors who are the least remote from the rest of the actors [22]. According to Bonacich [23], eigenvector is a good measure for determining the prestige or influence of an actor within their network. It can also be interpreted as how well-connected a given actor is to other well-connected actors. In this sense, prestige is a different measure to popularity; this difference comes from the fact that the popularity of an actor is a measure of the number of connections from other actors to this one, without taking into account whether these connections come from actors who are not popular themselves. Prestige, on the other hand, takes into account the characteristics and SNA metrics of the actors that choose given connections.

The first comment from the teachers about student prestige is to stress the difference between prestige inside the classroom and outside the classroom. Teachers tend to consider those students with greater intellectual capabilities as most prestigious (in the classroom environment), while outside the class, prestige is more related to level of family affluence or participation in “popular” activities.

### 3.5. Results Regarding Friendship

There are a number of different definitions of the concept of friendship [24,25,26]. In this study, the definition from [25] has been adopted, according to which friendship exists without the need for reciprocity in the relationship.

This concept raised great controversy among teachers. When they were asked about how and why friendship relationships came about, there was no consensus about the main cause. While some of them thought that students needed friends as partners to trust, others declared that satisfaction of personal interests was the main reason.

Teachers from the first grade stated that the closer friends of their students were outside the class and even outside the school, because they had just arrived there from other geographical locations. By the second grade, students have built a denser friendship network with their classmates.

An important distinction was also made when talking about secondary schools in rural areas. Rural students usually go to schools that are located in a village different from the one where their home is. In such cases, the strongest friendship relationships are found in the localities where they live. Something similar was pointed out in relation to students who live far from the school’s location, despite living in the same city.

### 3.6. Results Regarding Addictions and Substance Consumption

Teachers agreed that alcohol is the most widely consumed substance among adolescents. Binge drinking was also pointed out as a usual behaviour. Adolescents from the same class, and even those from other classes or from other schools, regularly meet up outdoors in order to drink together. Even during school hours, alcohol consumption takes place—for example, during study breaks—and it is quite alarming to realize that students do not attach importance to this fact.

Some teachers affirmed that they were able to notice the effect of binge drinking over the weekend by observing student behaviours on Mondays. The same could be applied to Fridays in relation to drinking on Thursdays.

The teachers also agreed, as do some studies, about the difficulty that students have in grasping the consequences—in both the short-term and long-term—of alcohol consumption disorders, and they stressed the relevance of other substances such as tobacco and cannabis (in fact, some teachers also reported the effect of cannabis consumption on some students, and the influence of this consumption on their behaviour and on the rest of the class).

## 4. Discussion

This study has raised a number of interesting issues regarding the use of social network analysis (SNA) for analysing student relationships in secondary school environments. Results from the study have a number of implications concerning the different phases of a SNA study and its application. 

Performing an SNA study starts with the careful design and construction of a questionnaire to be completed (in this case by the students) in order to obtain significant information about the individual characteristics and the social relationships in which the actors are involved. Posing the right questions will help to determine individual positions and roles in the context of these social networks.

This study has revealed a number of facts to bear in mind before obtaining student data. A first outcome of the study indicates the importance of taking into account the differences between the first and the second grades of secondary school, and the different particularities that distinguish them. This applies to a number of the studied items, and especially to sociability, approachability, popularity, closeness, and friendship. These results are in line with the existence of different interests sought by adolescents as they evolve over time [27].

The opinions of teachers about the relationship between popularity and nonconformity echo some of the findings from studies such as that by [28]. However, it is important to stress that this concordance is clearly stated by teachers only for second grade students, while attractiveness and style must be taken into account for finding popular students in the first grade.

Results about pro-sociality and intermediation also have some similar findings, described in works such as [29], where the importance of values as principles for guiding behaviour and relationships for the adolescent is also stressed. Teachers related popularity to family affluence, which is in line with the results from studies such as [30], but they also stressed the importance of participation in activities which are seen as “popular” (for example, being good at sports).

Another important issue arises if the geographical areas where students socialize and live are taken into account. This spatial consideration greatly affects, for example, the concept of “prestige”, friendship relationships, and substance consumption habits. A crucial distinction is also found when the “inside the classroom” and “outside the classroom” contexts are considered. A good SNA study would have to take these facts into account. For example, it is very important to know if the majority of friendship relationships are established inside the classroom or in other environments. These results are consistent with traditional studies about friendship in adolescents that include geographical considerations [31].

Once a questionnaire to obtain data has been formulated and then completed by students, it is also important to take into account the previous considerations when drawing conclusions from the SNA analysis process. For example, a given student could not be tagged as “lonely” without taking into account whether they are in the first or in the second grade, if they live far from the school, or even if they have a greater number of friends outside the classroom. As another example, the concept of “prestige” should also be contextualized, and a distinction should be made between when it is used in an “inside the classroom” environment and when it is used in an “outside the classroom” context.

The teachers’ perceptions regarding the consumption of alcohol (and other substances) deserve separate consideration. There is a great deal of concern among teaching staff about alcohol and substance consumption levels and, more worryingly, about the lack of knowledge about the mid- and long-term effects of this consumption. Similar considerations have also been pointed out in different, previous works [32,33,34].

The teachers also agreed—as do some other studies [35]—about the difficulty that students have in grasping both the short-term and long-term consequences of alcohol consumption disorders. In the case of alcohol, it is also worth noting that previous studies suggest that drinking is socially rewarded and associated with social status or popularity among adolescents [36,37].

Teachers also reported the existence of a relationship between the consumption of alcohol and the use of other substances such as tobacco and cannabis, both of which are easily accessible for students. This statement is in line with the results of a study [32] in which a similar conclusion was presented.

**Figure 2:**
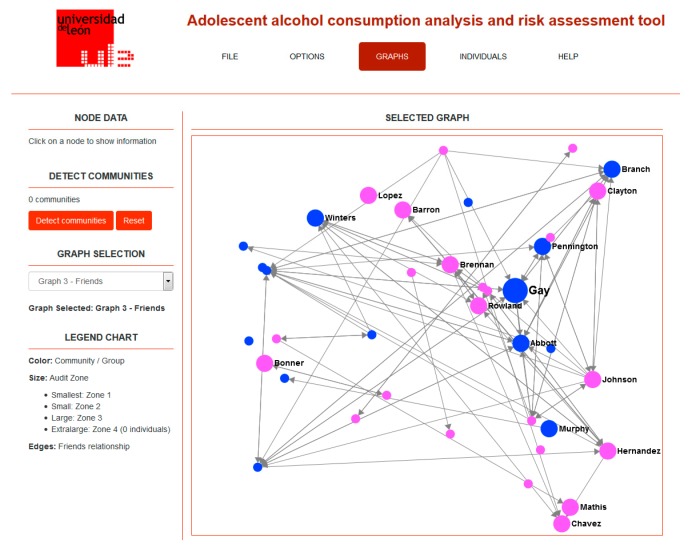
Screenshot of the application showing a friendship network for a given class (anonymized names).

**Figure 3:**
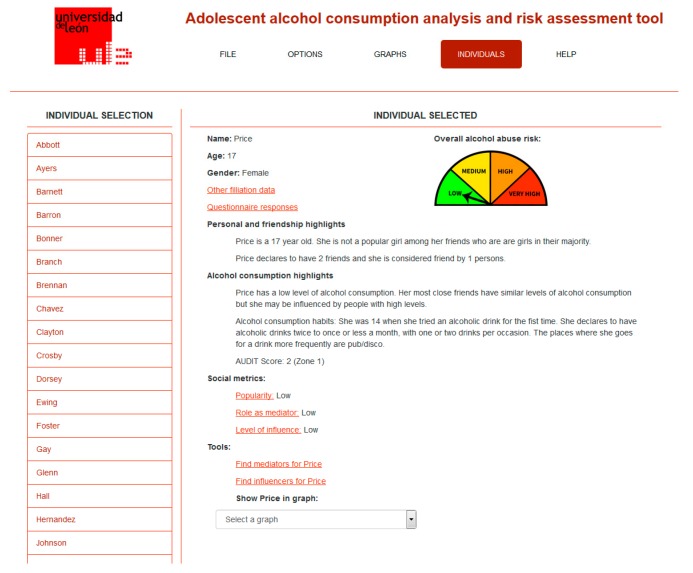
Screenshot of the application showing the results of the SNA analysis for an individual.

Some of the initial findings of this study have been used to design and build a computer tool for studying social relationships among adolescents and their alcohol consumption habits [38]. This tool includes automated data gathering (from the questionnaire design to its completion by the students), analysis, and visualization. The application is designed in such a way that someone who is not an expert on social network analysis can still obtain valuable information about the roles, positions, and relationships that are established among adolescents, as well as their alcohol consumption levels and risks. This is possible thanks to the use of semantic mappings between terms from SNA and usual interpretations expressed in everyday language. Figure 2 shows a screenshot of the application, where the friendship social network for a given classroom is displayed. In Figure 3, the information for an individual student is shown, where natural language descriptions are built from SNA analysis results and presented to the user, avoiding the use of SNA-specific terminology.

### Limitations and Future Work

The limitations of this study stem from the contextualization of the results and the limited number of teachers who were able to attend the working sessions. These factors make it difficult for the results to be generalized. Further work is needed in order to do so, but these initial outcomes are promising and open a number of research possibilities.

Future work in this area may include comparing teachers’ perceptions of student roles and positions with the findings from the SNA analysis in order to improve the previously presented software tool. One step further would be to design and execute an intervention focused on alcohol (or other substance) consumption and that involves teachers and the help of SNA techniques by incorporating new capabilities into the software tool.

## 5. Conclusions

The objective of this study was to ascertain and describe teachers’ perceptions about student relationships in the classroom and their understanding of social network analysis measures relating to the adolescent social network, paying special attention to friendship and alcohol consumption habits. A number of the teachers’ perceptions are in accordance with studies and interpretations from social network analysis (SNA) measures, but a number of difficulties and different points of view have also been found, as described in Section 4.

The results from this study can be used, in the context of an SNA study, for questionnaire design and construction, and for the later processing of data. Moreover, these results stress that in order for it to be possible to use SNA techniques to help teachers when performing a given intervention related to substance consumption, there is a clear need to introduce teachers to the outcome of these techniques in a manner that is compatible with their perceptions and their customary use of everyday language. In this sense, the use of computer tools able to automate the gathering, processing and visualization of results obtained from the SNA analysis is seen as a necessity, and can be the success factor enabling teachers to benefit from the outcomes of these techniques.

## Figures and Tables

**Table 1:** Social network analysis (SNA) metrics and questions for the teachers.

Category	Common Interpretation	Questions to Be Put to the Teachers
Outdegree	Sociability or approachability	What are the characteristics of the most sociable/approachable students in the classroom?
Indegree	Popularity	What are the characteristics of the most popular students in the classroom? What are the characteristics of the most popular students in the social environment?
Incloseness	Closeness to the rest of the students	What are the characteristics of the students who display more closeness to the rest of their class?
Betweenness	Intermediation, pro-social nature	What are the characteristics of the students who seek benefits for the entire classroom?
Eigenvector	Prestige, influence	What are the characteristics of the students who have the highest prestige in the classroom? What are the characteristics of the students who have the highest prestige in the social environment?
Friendship	Friends	What kind of students have the greatest number of friends in the classroom?
		Do students have a greater number of friends inside or outside the classroom?
Addictions	Substance consumption and addiction risk	How do you perceive consumption of alcohol and other substances?
		How do different substances relate to addiction?

## References

[1] Kelman H.C. (2006). Interests, relationships, identities: Three central issues for individuals and groups in negotiating their social environment. Annu. Rev. Psychol..

[2] Magid V., Moreland A.D. (2014). The role of substance use initiation in adolescent development of subsequent substance-related problems. J. Child Adolesc. Subst. Abuse.

[3] Faggiano F., Vigna-Taglianti F.D., Versino E., Zambon A., Borraccino A., Lemma P. (2008). School-based prevention for illicit drugs use: A systematic review. Prev. Med..

[4] Osgood D.W., Ragan D.T., Wallace L., Gest S.D., Feinberg M.E., Moody J. (2013). Peers and the emergence of alcohol use: Influence and selection processes in adolescent friendship networks. J. Res. Adolesc..

[5] Duncan G., Boisjoly J., Kremer M., Levy D., Eccles J. (2005). Peer effects in drug use and sex among college students. J. Abnorm. Child Psychol..

[6] Bangura A.K. (2006). Teachers’ Strategies in the identification, change and retention of deviant students teachers’ strategies in the identification, change and retention of deviant students. Interdiscip. Soc. Work.

[7] Bolkan S., Goodboy A.K. (2009). Transformational leadership in the classroom: Fostering student learning, student participation, and teacher credibility. J. Instr. Psychol..

[8] Faggiano F., Minozzi S., Versino E., Buscemi D. (2014). Universal school-based prevention for illicit drug use. Cochrane Database Syst. Rev..

[9] UNODC School-Based Education for Drug Abuse Prevention. http://www.unodc.org/pdf/youthnet/handbook_school_english.pdf.

[10] Valente T.W., Pumpuang P. (2007). Identifying opinion leaders to promote behaviour change. Health Educ. Behav..

[11] Grunspan D.Z., Wiggins B.L., Goodreau S.M. (2014). Understanding classrooms through social network analysis: A primer for social network analysis in education research. CBE Life Sci. Educ..

[12] Maya-Jariego I., Holgado D. (2015). Network analysis for social and community interventions. Psychosoc. Interv..

[13] Ennett S.T., Bauman K.E., Hussong A., Faris R., Foshee V.A., Cai L., DuRant R.H. (2006). The peer context of adolescent substance use: Findings from social network analysis. J. Res. Adolesc..

[14] Reifman A., Watson W.K., McCourt A. (2006). Social networks and college drinking: Probing processes of social influence and selection. Personal. Soc. Psychol. Bull..

[15] Lozares C. (1996). La teoría de redes sociales. Pap. Rev. Sociol..

[16] Brandes U., Robins G., McCranie A., Wasserman S. (2013). What is network science?. Netw. Sci..

[17] Jariego I.M., Ramos D.H. (2015). Análisis de redes sociales e intervención comunitaria. Psychosoc. Interv..

[18] Costenbader E., Valente T.W. (2003). The stability of centrality measures when networks are sampled. Soc. Netw..

[19] Wasserman S., Faust K. (1994). Social Network Analysis: Methods and Applications.

[20] Freeman L.C. (1978). Centrality in social networks conceptual clarification. Soc. Netw..

[21] Freeman C. (1991). Networks of innovators: A synthesis of research issues. Res. Policy.

[22] Hanneman R.A. (1998). Introduction to Social Network Methods.

[23] Bonacich P. (1987). Power and centrality: A family of measures. Am. J. Sociol..

[24] Ali M.M., Amialchuk A., Rizzo J.A. (2012). The influence of body weight on social network ties among adolescents. Econ. Hum. Biol..

[25] Mulassi A.H., Borracci R.A., Calderon J.G.E., Vinay P., Mulassi M. (2012). Social networks on smoking, alcohol use and obesity among adolescents attending a school in the city of Lobos, Buenos Aires. Arch. Argent. Pediatr..

[26] Reiter-Purtill J., Ridel S., Jordan R., Zeller M.H. (2010). The benefits of reciprocated friendships for treatment-seeking obese youth. J. Pediatr. Psychol..

[27] Simon B., Mummendey A. (1997). Selbst, identität und gruppe: Eine sozialpsychologische analyse des verhältnisses von individuum und gruppe. Identität Verschied..

[28] De La Haye K., Robins G., Mohr P., Wilson C. (2011). Homophily and contagion as explanations for weight similarities among adolescent friends. J. Adolesc. Health.

[29] Barni D., Danioni F. (2016). Adolescents’ basic personal values and sense of coherence. Personal. Individ. Differ..

[30] Lucio E., León I., Durán C., Bravo E., Velasco E. (2001). Los sucesos de vida en dos grupos de adolescentes de diferente nivel socioeconómico. Salud Ment..

[31] Tyrrell N., Harmer N. (2015). A good move? Young people’s comparisons of rural and urban living in Britain. Childhood.

[32] Pérez-Milena A., de Redondo-Olmedilla M.D., Martínez-Fernández M.L., Jiménez-Pulido I., Mesa-Gallardo I., Leal-Helmling F.J. (2017). Cambios en el consumo alcohólico de riesgo en población adolescente en la última década (2004–2013): Una aproximación cuanti-cualitativa. Aten. Primaria.

[33] Teunissen H.A., Kuntsche E., Scholte R.H.J., Spijkerman R., Prinstein M.J., Engels R.C.M.E. (2016). Friends’ drinking norms and male adolescents’ alcohol consumption: The moderating role of performance-based peer influence susceptibility. J. Adolesc..

[34] Ali M.M., Amialchuk A., Nikaj S. (2014). Alcohol consumption and social network ties among adolescents: Evidence from add health. Addict. Behav..

[35] Golpe S., Isorna M., Barreiro C., Braña T., Rial A. (2017). Consumo intensivo de alcohol en adolescentes: Prevalencia, conductas de riesgo y variables asociadas. Adicciones.

[36] Gommans R., Müller C.M., Stevens G.W.J.M., Cillessen A.H.N., Ter Bogt T.F.M. (2017). Individual popularity, peer group popularity composition and adolescents’ alcohol consumption. J. Youth Adolesc..

[37] Moody J., Brynildsen W.D., Osgood D.W., Feinberg M.E., Gest S. (2011). Popularity trajectories and substance use in early adolescence. Soc. Netw..

[38] Benítez J.A., Labra J.E., Quiroga E., Martín V., García I., Marqués-Sánchez P., Benavides C. (2017). A web-based tool for automatic data collection, curation, and visualization of complex healthcare survey studies including social network analysis. Comput. Math. Methods Med..

